# Measuring the Effect of Visual Exposure and Saliency of Museum Exhibits on Visitors’ Level of Contact and Engagement

**DOI:** 10.3390/bs8110100

**Published:** 2018-10-28

**Authors:** Linda Nubani, Alyssa Puryear, Kristy Kellom

**Affiliations:** School of Planning, Design & Construction, Michigan State University, 552 W Circle Dr, East Lansing, MI 48824, USA; puryeara@msu.edu (A.P.); kellomk@msu.edu (K.K.)

**Keywords:** wayfinding, space syntax, visibility graph analysis VGA, museum layout, exhibit spatial location

## Abstract

This paper examines visitors’ movement patterns at the Broad Museum designed by Zaha Hadid. Characterized with free, open, and generally unbound spaces, visitors explore a curated exhibition at their own pace, route, and agenda. Unlike most other public environments, a museum lends visitors greater choice and control, and does not hold the social or spatial expectations of other facility types that might subject the visitor’s path of travel. In this study, 72 visitors were observed. A space syntax-based visibility graph analysis (VGA) was then performed to compute the visibility exposure and the spatial position of each exhibit within the museum. Negative binomial regression was used to look at the effects of spatial variables on visitors’ wayfinding, contact, and engagement with the pieces. Results showed that both the amount of visibility area around each exhibit, and its spatial position measured using space syntax techniques explained why visitors established a contact with the piece and their wayfinding behavior. Interestingly, however, the saliency of exhibits along with spatial variables were both strong predictors for why people arriving in groups split to engage with that particular exhibit. The simulation used in this study could be useful in curatorial decisions.

## 1. Introduction

### 1.1. Movement Patterns in Museums

There is a growing interest in studying the positioning of exhibits in museums. User function of a museum is quite unique from other interior spaces, as visitors often inhabit the space for a short time and roam about freely with the primary purpose of viewing objects within the space [1]. In their work on visitors’ movement in art galleries and exhibitions, Wineman and Peponis [2] introduced a third category of movement that people follow in museums. They called it “spatially guided movement”. They believed that the structure of layouts has an impact on visitors’ movement choices, whereby certain exhibits might be visited more often others.

In addition to the importance of the structure of the layout, increased awareness of the characteristics which impact visitor movements within a museum, also known as visual saliency, is crucial for a curator designing future exhibitions [1]. Visual saliency refers to cues or characteristics which attract or hold a viewer’s attention. Salience rating is the degree to which an object attracts human attention. Spatial decision-making of museum visitors may be impacted by two types of visual saliency: object-based and location-based [1]. Object-based salience is influenced by perception of visual attractiveness of an individual piece. For example, a piece with high salience rating may attract viewers to an area of the gallery which may otherwise be less frequently visited. The Mona Lisa, for example, is a good example of an artwork with high salience rating located far away from the entrance of the Louvre in Paris. Location-based salience is reliant on the physical position of the piece within the gallery, as specified by the curatorial team [1]. A work of art installed in a position with high visibility, such as spaces visible from multiple galleries or at the intersection of two galleries, may be noticed more frequently regardless of the object’s individual salience rating.

Some studies, however, suggested that our attention may not necessarily be impacted by the salience of the object, but more so by their location spatially. Janzen [3] ran three experiments on participants using recognition tasks, and conscious and unconscious memory processes to investigate wayfinding behavior. Objects were positioned at different locations. Using a virtually designed environment, these objects were masked in one experiment and not masked in the other. Results showed that objects along decision points were recognized faster than others.

Regardless of the existing environmental conditions and/or maps, signs, or other wayfinding methods employed within an art gallery setting, “visitors must still use their spatial abilities to orient themselves in space” [1]. To do so, visitors must seek out, process, and store information relevant to the perceived surroundings to execute a wayfinding task, even in the unrestricted form of exploratory movement experienced within a gallery space. The moment a visitor sets foot within a space and makes their first choice, cognitive information collection begins. From here, an initial mental map is constructed and is thereafter continuously adjusted as information of previous locations is stored and new information is presented, received, and reacted to. This acquired information becomes the visitor’s identifying characteristics of the spatial layout [4]. Such characteristics will typically fall into one of five fundamental categories—paths, nodes, landmarks, districts, and edges—as defined by Kevin Lynch to devise a practical mental map [5]. Lynch [5] also introduced the term legibility to describe how different environmental layouts contribute to the development of cognitive mapping. Although these principles were originally designed for urban and city scales to explore cognitive mapping, they can be applied to scales of interior spaces and act as key features in a visitor’s experience and memory [2].

### 1.2. The Use of Space Syntax in Studying Movement Patterns in Museums

There is growing interest among researchers for the use of advance techniques based on space syntax theory to evaluate the effect of spatial layouts on visitors’ movement within museums. Space syntax is a group of theories that examine the impact of spatial layouts on different human behavior. Since it was originated in the 1980s by Hiller and Hanson [6], many studies documented the impact of spatial properties on different types of behaviors, such as wayfinding in hospitals [1], employee performance in the workplace [7,8], or even preferences for selecting targets for crime [9]. Two space syntax techniques are worth noting here. The axial line analysis is a popular method in measuring the depth of spaces within a structure, regardless of distances traveled. Syntactically, a space is described as “integrated” if it can be reached from all the other spaces in the building easily by going through a small number of rooms. Similarly, a space is described as “segregated” if it can be reached by going through a large number of spaces first, regardless of the distance traversed. Another popular technique, an isovist-based technique, measures different qualities of visibility polygons from all vantage points within a space. In simple terms, an isovist is a polygon that is drawn to cover the amount of visible information 360 degrees around a vantage point [10]. In addition to the properties each visibility polygon has (e.g., area, perimeter, radial length, occlusivity, etc.), advancement in software now provides the ability to develop syntactic measures based on these visibility polygons by creating a grid of isovists at certain distances in a plan. This technique is known as visibility graph analysis (VGA), and results of this analysis include measures like visibility “integration” [11].

Dosen and Ostwald [12] examined the isovist properties of 24 variations they created for a virtual interior space in order to understand human perception of enclosure and exposure. The variations were the result of modifications in ceiling and roof angles, and window widths and heights, as well as the introduction of columns. An online survey was distributed to 159 participants from eight different universities. Interestingly, the authors found a strong positive correlation between plan isovist properties and respondents’ perception of exposure, and a strong negative correlation between plan isovist properties, along with the sum of sectional and plan isovist properties, and respondents’ perception of enclosure.

Li and Klippel [13] used the visibility graph analysis technique to explore wayfinding patterns at a library where people expressed difficulty navigating its layout. Eight participants were asked to seek certain books from three different sections of the library. Arrangements of book stacks within these sections created different layouts that ranged in complexity and visibility. When the researchers conducted visibility graph analysis on all three library sections, results showed that participants, regardless of their familiarity with the library, spent longer at the section characterized with lower visibility. Lee, Ostwald, and Lee [14] also used the visibility graph analysis technique by evaluating three pairs of residential aged care facilities in three different countries. Using these techniques allow one to observe cultural characteristics within different plans. For example, the VGA output of connectivity color-codes the plan from red (visually connected) to dark blue (least visually connected). The Korean VGA output, for example, showed that both buildings enjoyed a large volume of visibility from most locations, while the two Australian buildings showed a hierarchical pattern from visitors to nurses to residents.

Although movement patterns in museums are different from finding a book at a library, Tzortzi [15] also used space syntax techniques to capture these patterns. In a comparison of the structural layout and positioning of exhibits in two different museums, Sainsbury and Castelvecchio, Tzortzi [15] used space syntax to explain observed movement patterns of 100 visitors in both museums. Both museums were designed for permanent exhibits. The former, however, was designed to become a museum, while the latter was a historical building that was converted into a museum. Sainsbury museum is characterized with long, open vistas generated by two major axes that touch the periphery of the building. These two axes connect a series of open spaces via wide door openings. The second museum also has long vistas, but the views do not open into one another in the way the first museum does. One needs to approach the next space for the visitor to have maximum visibility of its content. In other words, a visitor may not anticipate what he or she will see ahead. Results of observations revealed how visitors in Sainsbury mostly occupied the left wing of the museum and not the major axis that marked the major spine of the structure. In space syntax terms, the most integrated part of the museum (the major axis) attracted one-fourth of the visitors. Interestingly, however, the local syntactic property “connectivity” was a strong predictor for visitors’ movement. This could be explained by visitors having trouble deciding on their route once they step inside the museum. Space syntax also showed that the overall layout of the museum was not “intelligible”. Intelligibility is a space syntax measure that results from correlating global measures, i.e., “global integration”, with local measures, i.e., “connectivity”. When the ratio is below 0.5, intelligibility is referred to as low, making navigation within the space a little more difficult. In the case of the second museum, the researcher created a justified graph, another space syntax technique, and found that the overall structure had a very deep tree form that created a single directional route making it easy for visitors to return to the starting point without going through spaces already visited.

In their review of the changes to the High Museum of Art in Atlanta, Zamani and Peponis [16] discussed the changes to the co-visibility of exhibits through the use of visibility graph analysis. The study compared results from VGA analysis of the exhibits during four different phases that took place at the museum. The first three phases documented changes to the layout of the museum. The 1983 layout was first designed by architect Richard Meier, while the 1997 layout took place after the Olympics, and the 2003 layout marked a return to one more aligned with the original 1983 layout. The fourth phase took place when the modern expansion designed by Renzo Piano in 2005 was added to the museum. One of the differences between the expansion and the original museum is that it has 40-foot-wide clearances as opposed to the 21-foot grid of columns in the 1983 original building. The 1983 and the 2003 layouts were based on the idea of having a room within a room concept, where visitors would experience constant changes in visual patterns. The 1997 layout, however, was marked with exhibits following the periphery of the original structure and the existing columns acting as the internal divisions. Results of VGA showed how the co-visibilities of objects were 77% for 1983, 56% for 1997, and 100% for 2003. The 2005 layout appears to be more open with higher visual integration within the entire plan. However, when compared with the adjacent 1983 plan, the latter appears to be conceived as a choreography of co-visibility, social co-presence, and co-awareness [16].

Wineman and Peponis [2] tested two traveling science exhibitions curated by Carnegie Science Center. Both exhibits were adapted to two different open-plan exhibition halls (singular spaces) to assess visitor behavior patterns in relation to the exhibition content (which remained constant in both settings) versus spatial variables. By testing the same exhibit in different environments, this experiment evaluates how significant spatial variables affect “exploratory movement, visual contact, and active engagement with individual exhibits”. To do this, visitors in both settings were randomly selected to have their movement and behaviors unobtrusively tracked and recorded with attention to their contact and engagement with pieces featured in the exhibition, how long they visited, what order they made contacts and engagements, and so on to later assign behavior performance scores to visitors. These behavior performance scores, space syntax analysis (overall accessibility, overall visibility, mean depth and area, integration, connectivity, and intelligibility), and principles of mental mapping were used in conjunction to quantify the environment/visitors’ behaviors, to synthesize and calculate data, and to analyze the results for significant correlations to explain the data and justify the validity of hypotheses set forth.

Results of their experiment confirmed what previous experiments and literature proposed, such as informational spatial learning—the homeostatic process of becoming more comfortable and knowledgeable within an environment as the duration of stay increases. Furthermore, by comparing data of various methods, this study was able to confirm that “spatial variables produce more powerful effects on visitor behavior as the overall exploration time increases”. Lastly, this experiment was able to justify fundamental explanations for visitor behavior considering accessibility. In other words, more accessible pieces are more likely to have contacts, and more visible pieces will attract more engagement [2].

In a large study of museums, Tzortzi [17] analyzed layouts, visitors’ movement, and viewing rates in five large museums. The author used graph techniques from space syntax to better analyze the spatial configurations of these museums. He also used few variables based on observations: the tracking score gave the percentage of visitors in each space, while the mean tracking score for a museum showed whether visitors visited all spaces, and the tracking score differentiation showed how far spaces were visited. In Pompidou, Paris, where a main axis places galleries on both sides, movement was found globally structured but locally exploratory. The layout was highly hierarchical, where spaces led to other spaces, giving visitors multidirectional views. Although the plan is legible, visitors tended to miss parts of the museum. The Tate Modern in London also had a central axis; however, galleries were organized around its center. Although it is highly linear and people visited most of the spaces, there was a lot of backtracking generated as opposed to being exploratory. If one compares the main axis of these two museums, one finds that the axis brings people together at the Pompidou, while the axis at the Tate tends to distribute visitors to other galleries. The viewing rate at Tate was also noted to be different than the Pompidou, most likely because of the way exhibits were arranged.

More recently, Krukar and Dalton [18] used an eye-tracking device to track what 32 participants really saw at an art gallery. After the participants visited the space, they were given a printed layout along with thumbnail pictures seen inside the gallery. It is worth noting here that two conditions were created where the location of the pictures changed. The authors investigated the total fixation time within a picture, the percentage of time spent, and the number of dwells each object received. Using the same visibility graph analysis reported earlier, the authors found that, in both settings, the amount of visibility area (known as isovist area) and visual connectivity were high predictors for the number of dwells, simply because it was more likely to fall in sight. Correlations with total dwell time were significant, but lower. The authors also reported, in a different paper, that they found that salience rating did not explain total dwell time.

### 1.3. Research Objectives

The purpose of this research was to evaluate the ideas presented in space syntax literature in exploring the role spatial layouts play in art galleries to discover why people make various spatial decisions and why certain exhibits are visited more often than others. In addition to evaluating the impact of layouts on visitors’ movement choices, it also evaluates the impact of the salience each exhibit has on these spatial decisions. Two hypotheses were tested. The first hypothesis was that visitors to the museum are guided spatially through the gallery and establish contact with artworks that are visually integrated and enjoy a higher amount of visibility around them. The second hypothesis was that visitors most likely engage with art pieces that are both visually integrated and that are high in saliency, measured by the split factor.

Similar to the Wineman and Peponis [2] study, their concept of visitor contact and engagement was applied in this research to assist in quantifying and explaining visitor behavior. In this study, a contact was a brief encounter with a piece of art without actively making cognitive connections. For example, a short glance would be considered a contact. An engagement was defined as one’s willful cognitive involvement with a piece. Engagements could include actions that constitute interest, such as long gazes, discussing a piece with another visitor, reading a description, splitting from one’s own group, or taking a photo of a piece. Engagements could be contacts, but contacts could not be engagements. This study follows the methodologies followed in earlier work by Wineman and Peponis [2]; however, the authors also discuss the effects of the saliency of art pieces and their spatial locations on contact and engagement. The results of this study can be useful for curators as they make decisions in positioning their next exhibit.

## 2. Methodology

### 2.1. Case Study

This study was conducted at the Eli and Edythe Broad Art Museum, a contemporary art gallery and community resource located in East Lansing, Michigan. Designed by Zaha Hadid, the first woman to receive the Pritzker Prize for architecture, this angular building was opened in November 2012. The large, convoluted, geometric, metal-façade building drastically contrasts the traditional red-brick historic buildings surrounding the site, acting as a landmark on the university campus it serves. This remarkable building attracts a diverse range of visitors on a local, national, and global scale.

Seventy percent of the building’s 46,000 square feet supports an exhibition space for international contemporary art, which is curated by an in-house preparation staff. Other public amenities offered at the Broad include an outdoor patio, café, retail shop, and educational spaces. This study focused on the second floor, which consists of three galleries and a lobby, since this floor does not include spaces that impact people’s navigation behavior, such as cafés or retail shops.

The Broad Art Museum is popularly known for not containing any traditional, 90° angles. The design of the interior reflects the exterior architectural forms through a series of angled lines that each have a unique vanishing point, creating non-coplanar surfaces and intriguing forms. Materials most heavily used in the museum are casted concrete, metal, glass, and Corian solid surface. The color scheme applied involves monochromatic/neutral black, grays, and white.

The use of line and direction in this building poses an especially interesting case for wayfinding and navigation, since visibility is constantly changing for anyone finding their way through the galleries. To explore these ideas further, this study specifically focuses on the second-floor galleries of the Broad Art Museum during the Michigan Stories exhibition, displayed 18 November 2017 to 25 February 2018. Much of the work on display was bright, colorful, and proto-punk band style with easily identifiable imagery (direct images, no abstraction).

**Figure 1:**
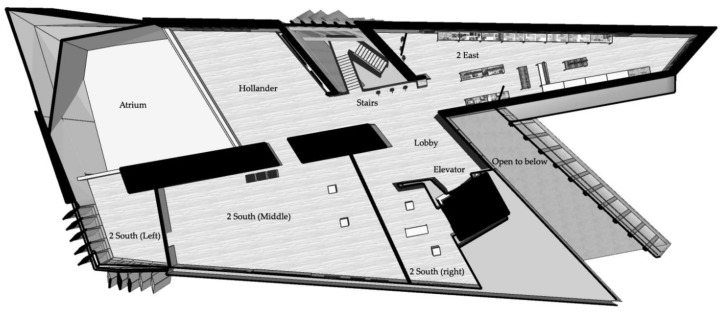
Isometric layout of the second floor of the Broad Art Museum.

The second floor of the Broad Art Museum consists of three main galleries: Two East, Two South, and Hollander Galleries (see Figure 1). The Two South Gallery was originally one large open space that was partitioned into three sections for this exhibit. These sections are herein referred to as Two South—Right (closest to the elevator), Two South—Middle, and Two South—Left. The second level can be accessed via staircase or elevator. It is important to note that the elevator has two doors (north- and west-facing) that either open to the Two South—Right Gallery or to a lobby area between the elevator and staircase. Therefore, depending on the user’s selection in the elevator, their initial understanding of the space could start at either of these points (see Figure 1).

Visible from the second floor are several architectural features (which could act as landmarks to visitors) and views to other floors and outdoors. Other floors are most prominently visible from the second floor at the floating staircase, a glass wall in the lobby overlooking a café on the first floor, and the balcony areas in Hollander and Two South—Left Galleries, between which is a void exposing the double-height Minskoff Gallery below.

As for wayfinding, there was no map provided to the user unless they took a small booklet from a stack on the reception desk on the ground level of the building, which contained a short description for each gallery’s contents. Signage on the second floor included code-required exit signs, emergency evacuation routes, elevator signage, and fire extinguishers. Other signage included that of gallery names near gallery entrances, installation names and descriptions near gallery entrances, informative labels about each piece, labels to deter guests from touching pieces, and donor recognition signage (staircase, lobby bench, Two South—Right bench). All gallery signage was in a bold/thick, sans-serif font, which was either black on a white background, or silver on a black background.

### 2.2. Observations

The authors followed the traditional visitors tracking system followed in museum studies and reports using a pen and a paper [19]. A total of 30 observations were carried out on four separate occasions on the second floor of the Broad Art Museum during the Michigan Stories exhibition. From these observations, a total of 72 visitors were observed (see Table 1). There were 22 observations that noted two visitors who walked in together, and four observations with three visitors who walked in together; the remainder of the observations noted a single case of one visitor who came alone, as well as cases of four visitors, five visitors, or six visitors. On each observation occasion, the date, time, and weather conditions were recorded. The first set of observations was conducted on Friday, 16 February 2018, and seven observations were collected. Eleven observations were done on the following day (Saturday, 17 February 2018). A third set of four observations were collected on Thursday, 22 February 2018. The remaining eight observations were completed on Sunday, 25 February 2018. Each observation day began in the early–midafternoon with comparable weather conditions.

The observer self-positioned at the lobby bench before each observation and tracked subjects in a discreet manner to not disrupt naturally occurring behaviors by not engaging with or revealing the observer to subjects in any way. The observer first recorded their point of access to the space (either staircase or elevator: north or west door) and what time they arrived. Then, a general user description was recorded which included the number of people in the group (if more than one) and their gender(s) and approximate age range(s). While subject(s) traveled through the galleries of the second floor, the observer logged their behaviors, movements, timing, viewing/gaze, and interactions with the space around them. To record these items, the observer utilized a table (to note time spent, subject actions, and additional notes) and a floor plan of the second floor (to track spatial route and navigation) for each observation. Finally, the observer recorded the point of exit (either staircase or elevator: north or west door) and the time the subject(s) left the space. These written observations were then converted to a digital format and used to enter data into an Excel sheet.

### 2.3. Visual Saliency

To calculate object-based salience rating, visual features such as intensity, color, and orientation were evaluated to determine each object’s visual appeal [20,21]. The intensity of an object factors in visual weight, complexity of visual depth, visual texture, contrast, and brightness. Color refers to variation of hue, shade, and value. Orientation of an object considers location and placement of the installation within a gallery, as well as size and scale, visibility, and attractiveness. Object-based salience ratings for each work of art on display during the Michigan Stories exhibition were assessed by the authors of this study using a system of high (very salient), medium (moderate saliency), and low (not salient). The rankings were cross-rated by two additional researchers to avoid bias. For example, an exhibit with high level of complexity and that was large in size may have received the rating of “high”, and an exhibit with minimal contrast that was small in size may have received the rating of “low”. The final ranking took the average ranking each exhibit received from all three researchers.

### 2.4. Visibility Graph Analysis

In addition to the role that the structure plays in how visitors perceive the spaces, the positioning of hanging art pieces may also add to the complexity. Therefore, two visibility graph analyses (VGAs) were performed on the second level of the museum using Depthmap developed by Turner et al. [22]. The first analysis was performed on the building layout as originally designed by Hadid. The second analysis treated the large hanging paintings as visual obstacles, which were, therefore, included in the analysis. According to literature, spaces with intelligibility values higher than 0.5 are more likely to impact wayfinding easily, while those with lower intelligibility values have a more challenging wayfinding experience [8].

In both scenarios, isovists were placed on a one-foot-interval grid. There were a total of 69 exhibits. The isovist area and the visual integration value for each exhibit were calculated (see Section 3).

## 3. Results

### 3.1. Observations

In this analysis, each exhibit had the following variables attached to it: visibility/spatial variables, number of visits it received, number of people who split from their group to engage with the exhibit, and the salience rating for that exhibit. Table 1 shows the number of visitors observed during the study.

### 3.2. Visibility Graph Analysis Output

**Figure 2:**
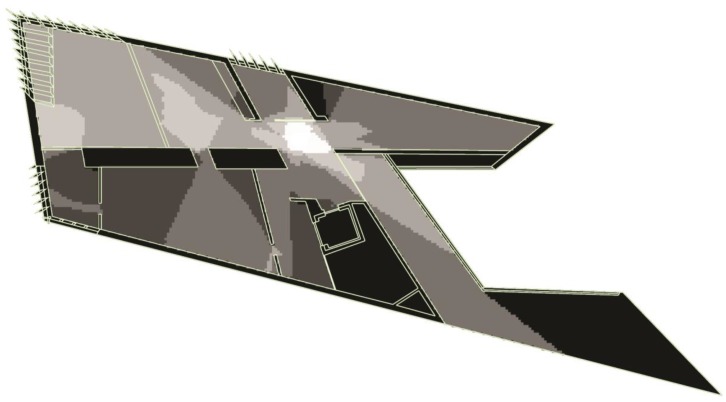
Visibility graph analysis (VGA) output of global integration at the Broad museum before the installation of the exhibits. Colors range from white (highly globally integrated) to black (least globally integrated).

**Figure 3:**
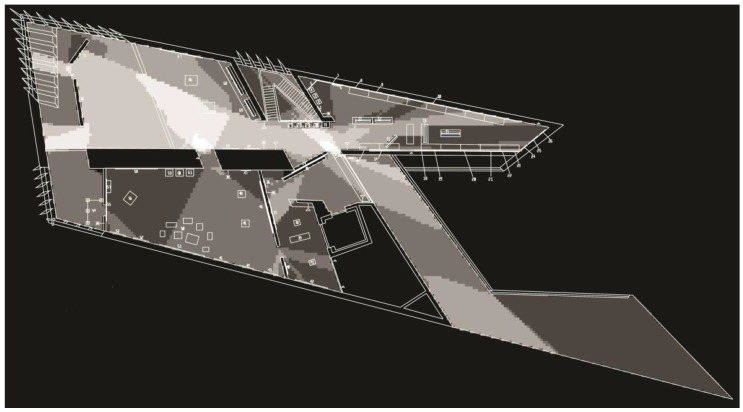
VGA output of global integration at the Broad museum after the installation of the exhibits. Colors range from white (highly globally integrated) to black (least globally integrated).

**Figure 4:**
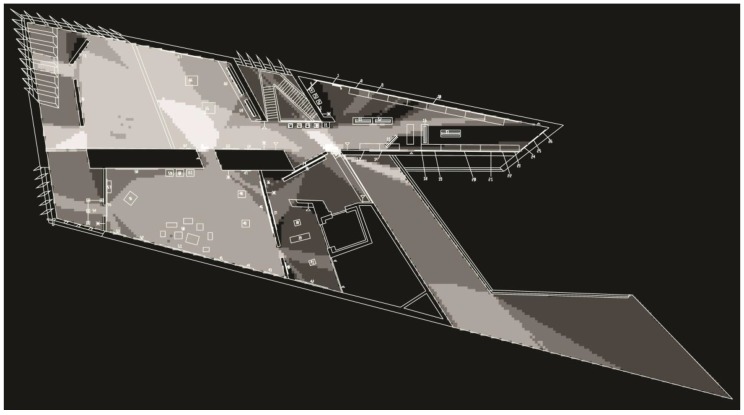
VGA output of local integration at the Broad museum after the installation of the exhibits. Colors range from white (highly locally integrated) to black (least locally integrated).

**Figure 5:**
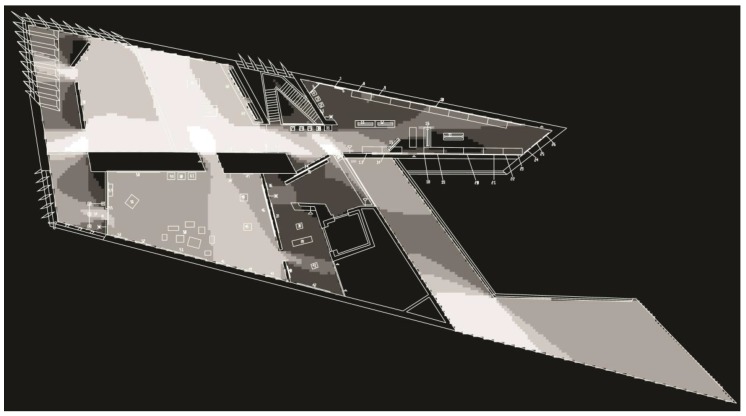
VGA output of isovist area at the Broad museum after the installation of the exhibits. Colors range from white (largest isovist areas) to black (smallest isovist areas).

When the intelligibility value was calculated for the analysis of the original gallery layout (see Figure 2), it produced a strong correlation between local measure and global measure (*R* = 0.75). The intelligibility value for the second analysis (see Figure 3 and Figure 4) that included the hanging artwork produced a lower value (*R* = 0.35). Although the authors did not explore the impact this difference has on visitors’ experience, it is worth noting that other studies demonstrated such links [8]. Results of visual properties showed that the isovist area within the second floor ranged between 25 and 4274 square feet with an average of 2120 square feet (see Figure 5). Exhibits, on the other hand, had an isovist area that ranged between 376 and 3400 square feet. The average visual field around exhibits was 1790 square feet. Visual integration for the exhibits ranged between 5.7 and 15.2 square feet.

### 3.3. Statistical Results

Pearson correlations between the spatial variables (global integration, local integration, and isovist area) and total visits each exhibit received were calculated. Table 2 shows that both local integration and isovist area were highly correlated with the total number of visits. Salience rating was not significant. The difference between global integration and local integration was that the former measured the number of visual steps from the exhibit, calculated from all the other isovists in the system, while the latter considered isovists that were few steps away. As Table 2 shows, visitors tended to make their spatial decisions based on the spatial location of the exhibit and the amount of area (exposure) around that exhibit.

To examine the nature of this relationship, a negative binomial regression was performed using the SPSS software. This test was selected because of the count nature of the data, and because the variances of independent variables were higher than their mean. In this test, it was important to drop one of the independent variables from the model due to the high collinearity that existed between local integration and isovist area (see Table 2 to see correlation value between isovist area and local integration).

Table 3 shows results from the regression model where, for every additional unit increase in local integration, that exhibit would be 1.7 times more likely visited than others. Results were significant at *p* = 0.000.

Another negative binomial regression was calculated to predict the number of separate visits that exhibits received from visitors from the same group, based on the salience rating of the exhibit (measured as low, medium, or high) and the spatial location of the exhibit (measured with global integration of isovists). The overall model was significant at *p* = 0.005. Table 4 shows results of the negative binomial distribution. Both the coefficient estimates and the exponentiated values of the coefficients are shown. To explain, exhibits with a salience rating of 1 received, on average, 0.8 fewer visits than exhibits with a salience rating of 3 (*p* = 0.008). Similarly, exhibits with a salience rating of 2 received, on average, 0.75 fewer visits than exhibits with a salience rating of 3 (*p* = 0.01). Additionally, for every additional unit increase in the integration value of an exhibit, that exhibit would be visited 1.15 times more often than others. Results were significant at *p* = 0.04.

## 4. Discussion

The results of this study demonstrate how powerful space syntax techniques are in understanding wayfinding behavior among museum visitors, and confirm the results of earlier work on visitors’ behavior in museums [2]. Simply put, the algorithm of this simulation software is built on the premise of mathematically calculating the amount of visible area at a grid of one-foot spacing, while exhibits, walls, and other above-eye-level obstacles act as boundaries. The output shows a range of colors, facilitating the evaluation of the spatial locations of each exhibit and allowing immediate recognition of which exhibits have larger visibility areas (in other words, higher exposure than others). Similarly, the same software has the capacity to calculate the number of visual steps one needs to make to reach a certain location or a certain exhibit. This calculation disregards distances out of the belief that one bases his or her decisions on what one sees. Unlike museums with permanent exhibits, it is the nature of this art museum to feature different exhibits periodically. The locations of new exhibits are the result of careful curatorial decisions. In light of the results of the simulations in this study, the authors will seek to simulate the spatial layout of different exhibits and further explore visitor’s wayfinding behavior, contact, and engagement with different exhibits. If visitation patterns were replicated, then the use of space syntax-based computer simulation may become an important tool in the positioning of exhibits at a museum. As shown in this study and other research work, space syntax studies may play a big role in cognitive science. One may use it as a tool to understand how configurational information of a space is mentally stored and utilized by people.

There were a few limitations noted in this study. Although it was interesting to note the high difference in intelligibility value of the overall space under the two different conditions (original layout vs. layout with hanging exhibits), the authors were not able to compare wayfinding under the two different circumstances, which may be worth exploring in future studies.

Further research should also control for factors like age and gender, in which different behavioral patterns may be detected, especially when it comes to engagement with exhibits. Another limitation to this study involved the computing of isovist properties on a plan level without taking into consideration the visibility volumes generated by a feature like an atrium. In other words, would an atrium within the second floor of the gallery have attracted visitors toward it? Could that be computed using the sectional isovist approach introduced in more recent studies by Reference [12]? The authors are also aware of the method that measures visual profiles along paths (also known as path isovists); exploring the order of these visits from a visibility perspective is worth examining.

## 5. Conclusions

The results of this research demonstrated the role of spatial location of exhibits in establishing contact with visitors using space syntax techniques, and the role of the salience quality of exhibits in engaging with visitors. It was also demonstrated that wayfinding behavior is impacted by the layout of the building, as well as the position of the exhibits within the spaces specifically if they impact visibility at an eye-level. Results of this study confirm earlier results reported in the literature, showing that current space syntax-based tools have the power to calculate the amount of visibility each exhibit has and how integrated (how easily accessible) each exhibit is. These two variables were important in guiding visitors through the spaces and, more importantly, in visiting certain exhibits while skipping others. Although the structure of the building is permanent, curators may always have the choice of altering the viewing sequence by hanging objects at eye level. However, using available simulation tools to conduct visibility graph analysis could be important in making their curatorial decisions, so as to ensure that all exhibits have a higher likelihood of being visited.

## Figures and Tables

**Table 1:** Number of visitors observed during the study (including number of visitors in a group).

Number of Visitors in a Group	Number of Observations	Subtotal
1	1	1
2	22	44
3	4	12
4	1	4
5	1	5
6	1	6
Total Observations	30	
Total Visitors		72

**Table 2:** Results from Pearson correlation between spatial variables and total visits.

Correlations					
		Local Integration	Isovist Area	Global Integration	Total Visits
Local Integration	Pearson Correlation	1	0.916 **	0.673 **	0.620 **
	Sig. (2-tailed)		0.000	0.000	0.000
	*N*	68	68	68	68
Isovist Area	Pearson Correlation	0.916 **	1	0.652 **	0.560 **
	Sig. (2-tailed)	0.000		0.000	0.000
	*N*	68	68	68	68
Global Integration	Pearson Correlation	0.673 **	0.652 **	1	0.176
	Sig. (2-tailed)	0.000	0.000		0.152
	*N*	68	68	68	68
Total Visits	Pearson Correlation	0.620 **	0.560 **	0.176	1
	Sig. (2-tailed)	0.000	0.000	0.152	
	*N*	68	68	68	68

** Correlation is significant at the 0.01 level (2-tailed).

**Table 3:** Results from negative binomial regression of total number of visits.

Parameter Estimates										
Parameter	B	Std. Error	95% Wald Confidence Interval		Hypothesis Test			Exp(B)	95% Wald Confidence Interval for Exp(B)	
			Lower	Upper	Wald Chi-Square	df	Sig.		Lower	Upper
Intercept	−2.644	0.8902	−4.389	−0.900	8.825	1	0.003	0.071	0.012	0.407
Local Integration	0.537	0.0956	0.349	0.724	31.539	1	0.000	1.710	1.418	2.063
Dependent variable: total visits Model: (intercept), visual local integration										

**Table 4:** Results from negative binomial regression of total number of split visits.

Parameter Estimates										
Parameter	B	Std. Error	95% Wald Confidence Interval		Hypothesis Test			Exp(B)	95% Wald Confidence Interval for Exp(B)	
			Lower	Upper	Wald Chi-Square	df	Sig.		Lower	Upper
Intercept	0.088	0.6425	−1.172	1.347	0.019	1	0.891	1.092	0.310	3.846
Salience rating = 1	−0.827	0.3105	−1.435	−0.218	7.089	1	0.008	0.438	0.238	0.804
Salience rating = 2	−0.752	0.3193	−1.378	−0.127	5.552	1	0.018	0.471	0.252	0.881
Salience rating = 3	0 ^a^							1		
Global integration	0.147	0.0738	0.002	0.292	3.960	1	0.047	1.158	1.002	1.339

Dependent variable: total split visits. Model: (intercept), salience rating, visual global integration ^a^ coeffecient B is zero because it is the reference group.
